# Dielectric and Electrical Properties of BLT Ceramics Modified by Fe Ions

**DOI:** 10.3390/ma13245623

**Published:** 2020-12-09

**Authors:** Beata Wodecka-Dus, Tomasz Goryczka, Małgorzata Adamczyk-Habrajska, Mateusz Bara, Jolanta Dzik, Diana Szalbot

**Affiliations:** Institute of Materials Engineering, Faculty of Science and Technology, University of Silesia in Katowice, 75 Pułku Piechoty 1a, 41-500 Chorzów, Poland; beata.wodecka-dus@us.edu.pl (B.W.-D.); tomasz.goryczka@us.edu.pl (T.G.); mbara1@us.edu.pl (M.B.); jolanta.dzik@us.edu.pl (J.D.); diana.szalbot@us.edu.pl (D.S.)

**Keywords:** BLTF, traditional ceramics, X-ray diffraction (XRD), dielectric properties, electrical properties

## Abstract

The solid solution of the perovskite type structure Ba_0.996_La_0.004_Ti_1−*y*_Fe*_y_*O_3_ (BLTF) for varying iron content (*y* = 0.1−0.4 mol.%) was obtained as a result of a solid state reaction using the conventional method. At room temperature (*Tr < T_C_*), the as-received ceramics reveals a single-phase, tetragonal structure and a *P4mm* space group. An increase in the iron content causes a slight decrease in the volume of the elementary cell. In addition, this admixture significantly reduces the maximum permittivity value (*ε_m_*) and the shift of the phase transition temperature (*T_C_*) towards lower temperatures. The BLTF solid solution shows a classical phase transition and low values of dielectric loss tangent (tg*δ*), both at room temperature and in the phase transition area. The Curie–Weiss temperature (*T*_0_) and Curie constant (*C*) were also determined on the basis of the dielectric measurements results. The analysis of temperature changes in DC conductivity revealed presence of the positive temperature coefficient of resistivity (PTCR) effect in the phase transition area.

## 1. Introduction

An important class of perovskite compounds are materials made on the basis of barium titanate. The interest in these materials has been continuous for several decades and is associated with the development of new applications in microelectronics, as well as in the industry of actuators, motors, and transformers. The colossal values of electric permittivity are obtained by adding the admixture of ions, characterized by small ionic radius, into the barium titanate structure. The admixture concentrations are small since they aim to change the polarizability, while at the same time, not significantly altering the BaTiO_3_ crystal lattice. The elements that meet the above-mentioned conditions are: iron, nickel, cobalt, magnesium, calcium, manganese, and lanthanum which cause the greatest changes in the effective values of electric permittivity [1]. As a result, barium titanate ceramics doped with lanthanum is the most attractive material from the point of view of potential applications. The colossal values of electrical permittivity, characterizing the ceramics of lanthanum titanate (BLT), are associated with the occurrence of electromechanical coupling [2]. However, the main attention of researchers is focused at the positive temperature coefficient of resistivity (PTCR) connected with the jump of few order in magnitude of the resistivity in a small temperature range above the ferroeletric–paraelectric transition temperature [3,4,5]. The PTCR effect is a grain-related phenomenon [6]. One of the most accepted theories to explain the temperature resistivity behavior above Curie temperature in BaTiO_3_-based materials is the Heywahgy model. The basic premises of this model are [7]:

- the PTCR relies on a grain-boundary effect,
- the grain-boundary permittivity follows the Curie–Weiss law and equals the permittivity of single crystals,
- the electron–trap layers are two-dimensional along the grain boundary. The dielectric properties of the layers are different than those of the bulk phase. Taking into account the influence of the polarization on the resistivity below the Curie point, proposed by Jonker, extended the premises [8].

In contrast to lanthanum, the admixture of iron induces ferromagnetic properties in BT and the occurrence of large leakage currents [9]. An additional advantage of barium titanate modification with Fe^3+^ ions is the fact that the occurrence of magnetic moments does not cause the disappearance of the ferroelectric properties of this compound [10]. Iron and barium titanate are two "classic" ferroic materials with very well-studied properties. Barium titanate, as already mentioned, is an oxide ferroelectric perovskite possessing a spontaneous polarization at the *P_S_* ≈ 0.26 C/m^2^ level and the Curie temperature *T_C_* ≈ 120 °C, in which the transition from the regular crystallographic system to the tetragonal one occurs. Iron, on the other hand, is a metallic ferromagnetic with a magnetic moment equal to 2.22 μ_B_ and Curie temperature *T*_C_ ≈ 770 °C [11]. An extremely important fact is that Fe (110) and BaTiO_3_ (100) have a very good match of lattice constants (at the level of 98.6%), which enables epitaxial growth of multilayered Fe/BaTiO_3_ layer by layer, without unmatched dislocations [12].

Our previous studies have been focused only on an undoped ceramic material, namely Ba_0.996_La_0.004_Ti_0.999_O_3_ (BLT4) [13]. The proper selection of synthesis conditions resulted in the creation of an appropriate concentration of donor levels and oxygen gaps, which in turn led to a significant increase in electrical permittivity, with small values of the dielectric loss factor. The results have shown that the material is promising for developing high performance ultracapacitors. Moreover, the appropriate level of material defects also affected the lack of reduction of ions mobility within the grain boundaries and the appearance of posistor properties within the temperature range of 120–170 °C. Due to their wide application possibilities, the BLT4 materials became a basis for subsequent studies of dopant influence on electrical properties.

In present study, based on previous experience, the BLT4 ceramics were modified with iron ions at an attempt to further improve the application properties. The new group of materials were studied in terms of their dielectric, structural, and microstructural properties. The influence of iron ions on temperature and other parameters of the phase transition from the ferroelectric to paraelectric phase are discussed in more detail. In addition, temperature changes in electrical conductivity are presented, on which the PTCR effect is revealed.

## 2. Materials and Methods 

**Figure 1:**
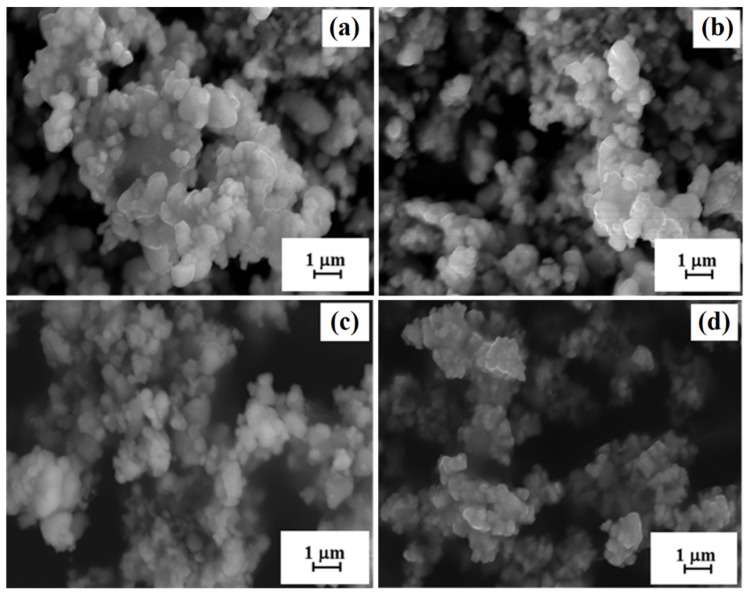
SEM images observed for ceramic powders: (**a**) BLTF1, (**b**) BLTF2, (**c**) BLTF3, and (**d**) BLTF4 after synthesis.

Fe_2_O_3_ iron oxide was added to the ceramic material with the perovskite type structure described by the following formula Ba_1−*x*_La*_x_*Ti_1−*x*/4_O_3_ for *x* = 0.4 mol.% (BLT4), thus a solid solution with the general formula of Ba_0.996_La_0.004_Ti_1−*y*_Fe*_y_*O_3_ (BLTF) was obtained, for *y* = 0.1−0.4 mol.%. On the basis of the general formula of the BLTF ceramics, the stoichiometric amounts of the following substrates: BaCO_3_, La_2_O_3_, Fe_2_O_3_, TiO_2_ necessary to obtain the planned compositions:

(1) Ba_0.996_La_0.004_Ti_0.999_Fe_0.001_O_3_ ⇒ BLTF1;
(2) Ba_0.996_La_0.004_Ti_0.998_Fe_0.002_O_3_ ⇒ BLTF2;
(3) Ba_0.996_La_0.004_Ti_0.997_Fe_0.003_O_3_ ⇒ BLTF3;
(4) Ba_0.996_La_0.004_Ti_0.996_Fe_0.004_O_3_ ⇒ BLTF4;

were calculated and weighed. As a result of wet mixing in a planetary ball mill, the solid solution of iron lanthanum barium titanate (BLTF) was obtained in the form of a microcrystalline powder. On the basis of thermal analyzes [14,15], the most favorable conditions for the synthesis of BLTF powders were *T* = 950 °C and process duration *t* = 6 h. After synthesis, the ceramic materials were crushed and milled again into powders. These powders were characterized by a homogeneous and—in terms of size—fine grain, which an average size did not exceed 1 μm. Due to the specific surface development, hard agglomerates and powder aggregates were formed (Figure 1).

Successively, the powders of each BLTF composition were sintered in the temperature range *T* = (1250−1350) °C, for *t* = 2 h. The relative density of discussed materials was determined with the Archimedes method. It changes from 5.68 g/cm^3^ for BLTF1 to 5.61 g/cm^3^ for BLTF4. 

The microstructure of the obtained ceramics was checked by means of a scanning electron microscope HITACHI S-4700 (SEM, Hitachi, Tokio, Japan). The crystal structure and phase composition of ceramic materials were studied at room temperature using the X’Pert PRO PANAlytical diffractometer (Philips, Amsterdam, The Netherlands). The X-ray diffraction patterns were measured in step-scan mode, in the 2*θ* angular range from 10° to 145°, with a step of 0.05° and a counting time of 10 s/step. The lattice parameters were calculated using Rietveld refinement implemented in the computer program—LHPM version 4.2. The goodness of the refinement was considered as a satisfactory when the values of mean reliability factors were lower than 8%.

The samples of cylindrical shape (0.6 mm thickness and 1 mm diameter) were prepared for the dielectric and DC measurements. Prior to the measurements, the ceramics were coated with platinum paste on both sides and annealed at 850 °C for 0.5 h to form platinum electrodes.

The dielectric properties of BT and BLTF ceramics were investigated using a computerized measurement system based on an Agilent E4980A LCR meter (Agilent, Santa Clara, CA, USA). Temperature characteristics of electric permittivity and dielectric loss were measured in several measurement field frequencies, selected from the range *f* = 0.1−1000 kHz, in the temperature range *T* = 20−210 °C. The measurements were carried out in both heating and cooling processes.

The measurement of the DC conductivity of the BLT ceramics doped with Fe^3+^ was carried out at a constant voltage of *U* = 10 V, in the temperature range 20−400 °C, using a measuring system, the integral part of which was a Keithley 6485 picoammeter(Tektronix U.K. Limited, Bracknell, UK) with contacts to the samples within a high temperature furnace. The temperature was measured using a LakeShore Model 335 (Lake Shore Cryotronics, Westerville, OH, USA) controller via a K-type thermocouple mounted directly on the insulation (alumina) of the ground electrode of the sample fixture. The picoammeter and temperature controller were interfaced with computer to collect data during cooling and heating processes at a rate of 5 °C/min.

## 3. Results and Discussion

**Figure 2:**
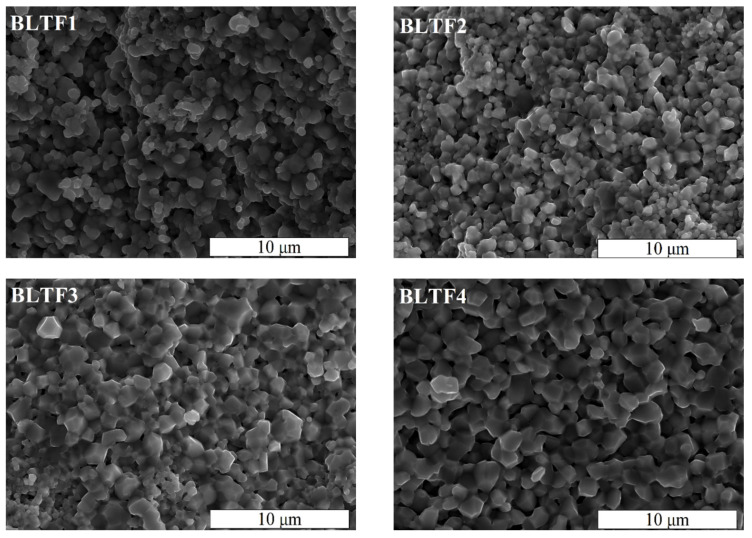
SEM images of BLTF1, BLTF2, BLTF3, and BLTF4 ceramics (5000× magnification).

Figure 2 shows the scanning electron micrographs of Ba_0,996_La_0,004_Ti_1-*y*_Fe*_y_*O_3_ ceramics with Fe^3+^ concentration in the range 0.1 ≤ *y* ≤ 0.4 mol.%, performed on the fractured surface of the ceramics at room temperature, at 5000× magnification. The surface of the fracture goes along the boundaries between grains, which is typical for material with large, well-shaped, hard grains. The microstructure of investigated samples shows a good homogeneity. Presented images clearly show the densely-packed, fine-grained microstructure. The grains are well developed and have angular shape. The images reveal the iron admixture caused increasing of average grain size from 0.5 μm for BLTF1 to 2 μm for BLTF4 (Figure 2).

**Figure 3:**
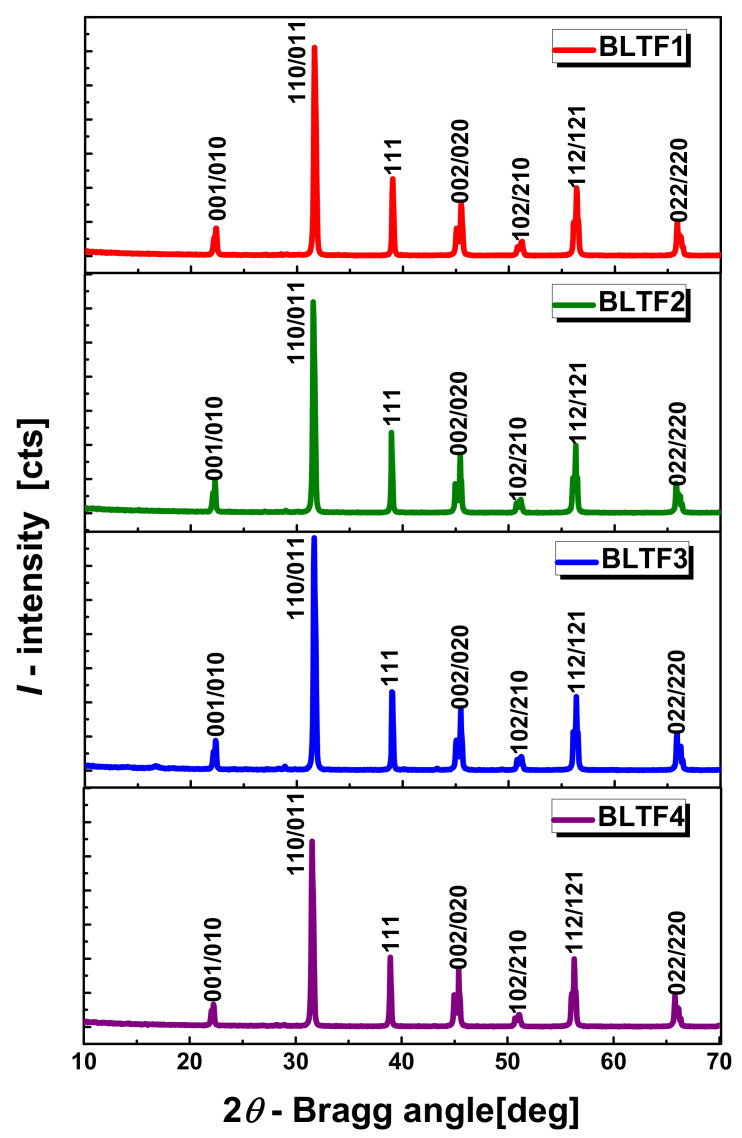
X-ray diffraction patterns measured at room temperature for BLTF ceramics.

The crystal structure, as well as qualitative and quantitative identification of the BLTF ceramics phases obtained by the conventional method were investigated by X-ray diffraction. The obtained diffraction reflections and calculated values of *d_hkl_* correspond to the values of the perovskite standard. Small deviations in the intensity and the position of diffraction lines can be attributed to the ceramic texturing occurring as a result of the pressing process (Figure 3).

The research reveals that the applied sintering conditions (*T* = 950 °C/6 h) favor the synthesis of BLTF material, which is confirmed by the formation of a single-phase crystal structure with tetragonal symmetry (*a*_0_ = *b*_0_ ≠ *c*_0_, α = *β* = *γ* = 90°) corresponding to the space group *P4mm* (No. 99). The received ceramics show the perovskite type structure ABO_3_, where the A octahedral positions are occupied by Ba^2+^ ions (with an ionic radius *r* = 1.35 Å) and La^3+^ lanthanum ions (*r* = 1.15 Å), whereas the B octahedral positions are occupied by titanium ions Ti^4+^ (*r* = 0.605 Å) and Fe^3+^ iron (*r* = 0.645 Å), with similar ionic radii [16]. The values of lattice parameters calculated from the Rietveld refinement are collected in Table 1.

As the concentration of iron increases, the volume of the elemental cell slightly decreases. This fact confirms an expected atom distribution in the crystal lattice. In addition, the reduction of the cell parameter may be related to the ordering of the crystal structure and lowering of the density of structural defects. It is known that oxygen vacancies cause a decrease in the Columbian attraction force between the anions and cations in the immediate vicinity of the elementary cell, which leads to an increase of interatomic distances. Thus, an increase in the volume of the elemental cell of the compound may be observed. Therefore, the small decrease of the lattice parameters and volume with the increase in iron concentration may be associated with a decrease in the concentration of oxygen vacancies.

The most important characteristic in the research of dielectric properties of materials is the temperature dependence of electric permittivity, allowing to determine the temperature of phase transitions occurrence and gives some information about its type. In turn, the temperature characteristics of the dielectric loss allow to draw conclusions about the loss of the discussed materials.

**Figure 4:**
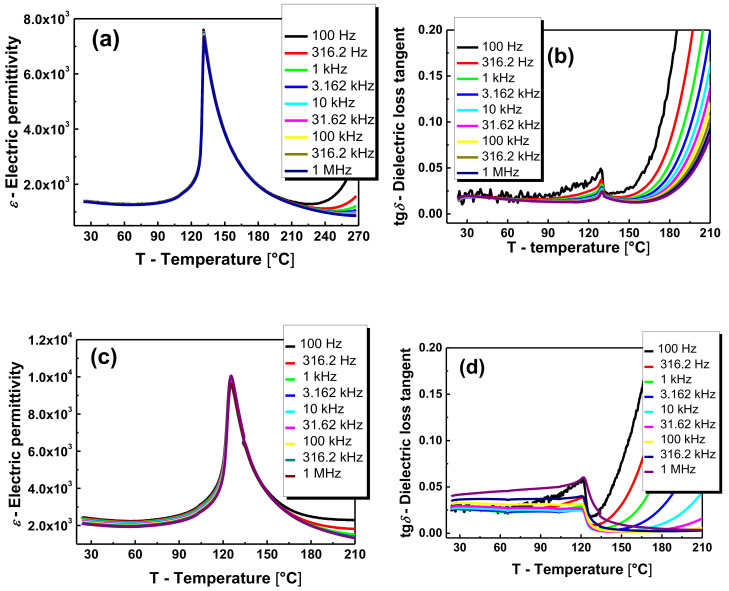

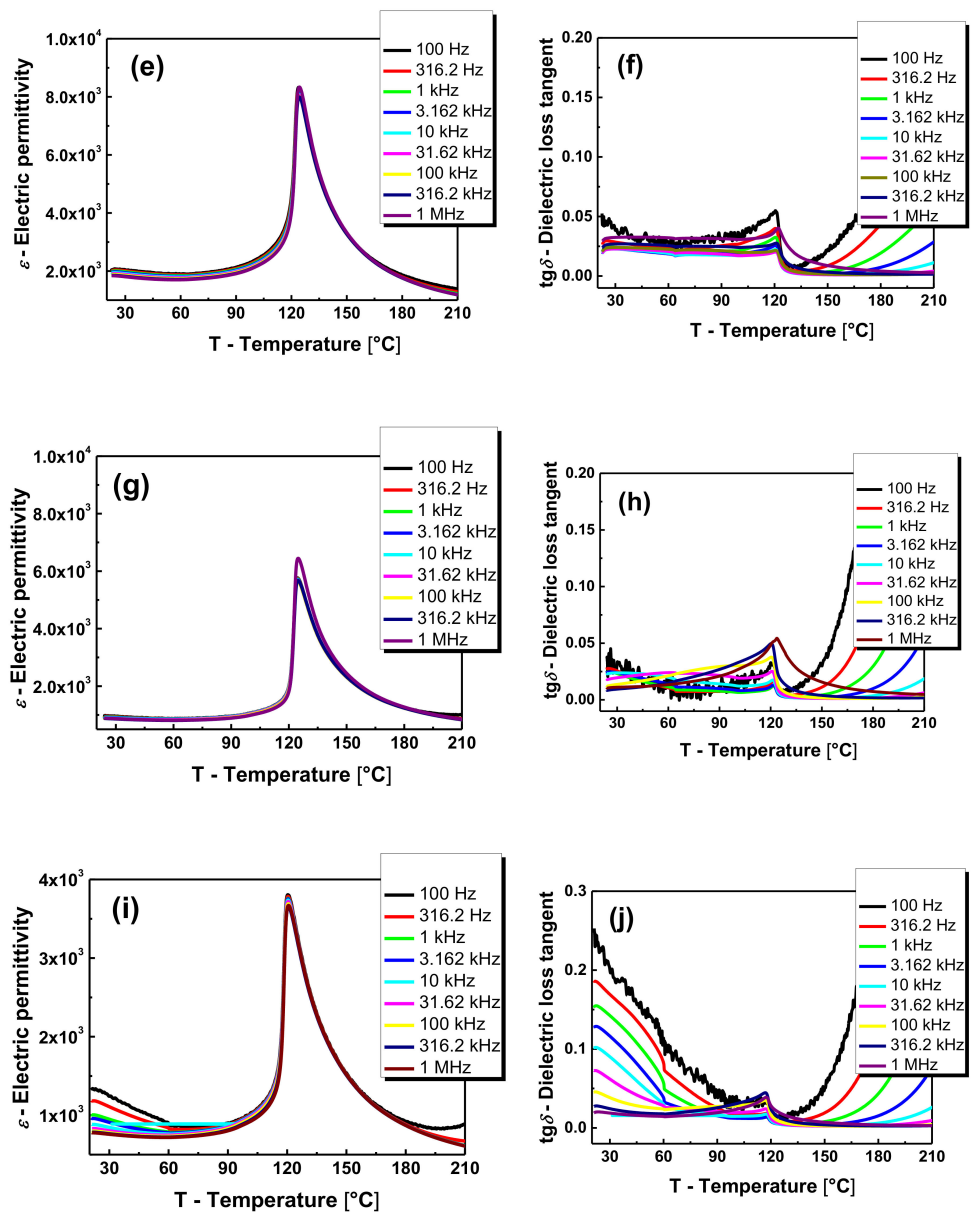
Temperature dependence of electric permittivity *ε* and dielectric loss factor tg*δ* measured at various frequencies of the measuring field for (**a**,**b**) BT, (**c**,**d**) BLTF1, (**e**,**f**) BLTF2, (**g**,**h**) BLTF3, (**i**,**j**) BLTF4 ceramics.

Figure 4 presents the characteristics of *ε*(*T*) and tg*δ*(*T*) of the BT and BLTF samples at different frequencies of the measuring field (from *f* = 100 Hz to *f* = 1 MHz) in the temperature range of 20−210 °C. Due to the high similarity of characteristics obtained in the heating and cooling process, solely the characteristics for the heating process are provided.

The dielectric permittivity of BaTiO_3_ ceramics at room temperature (ε_r_) equals 1350 (Figure 4a). The addition of 0.4 mol.% of lanthanum caused an increase of dielectric permittivity at room temperature up to ~39,500 as well as the increase of maximum value at Curie temperature [13]. The iron modification caused deterioration of dielectric properties. The breakdown is significantly visible in the case of iron concentration equal to 0.1 mol.%. The further increase of admixture concentration does not cause such profound changes (Figure 4c,e,g,i). (Table 2). The rapid decrease in electric permittivity can be explained by the introduction of larger iron ions into the crystal lattice instead of titanium ions with smaller radii. Such modification is connected with the decrease in the space for off centering [17]. Moreover, the lower valence of Fe^+2^ in comparison to Ti^+4^ created an oxygen vacancy leading to a break in the cooperative vibration of the Ti–O chains [18,19]. It can also be assumed that the inhibition of rapid changes in the case of higher dopant concentrations is related to the partial substitution of iron ions for much larger barium ions. The obtained BLTF ceramics is characterized by low values of the dielectric loss tangent, both at room temperature and in the range of phase transition. However, an increase in tg*δ* is observed in the area of the paraelectric phase, which is associated with an increase of the electrical conductivity. Moreover, the temperature dependencies of tg*δ* (*T*) measured at different frequencies of the measuring field point at significant relaxation. The origin of the phenomena is different at low and high temperature range. At low temperature range, it is probably associated with interaction between oxygen vacancies and ferroelectric domain walls. The mechanism is present only in the ferroelectric phase [20,21]. At high temperature range, the observed relaxation is connected with oxygen vacancies, the motion of which became independent and long range. Charges trapped in grain boundaries give rise to space charge polarization and observed relaxation [22]. 

**Figure 5:**
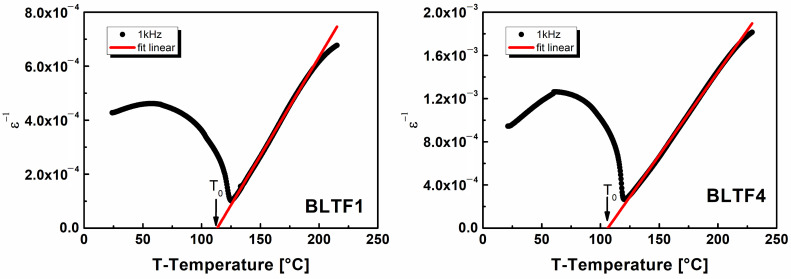
The reciprocal permittivity at 1 kHz as a function of temperature for BLTF1 and BLTF4 ceramics.

In the wide temperature range of the paraelectric phase in BLTF ceramics, the 1/*ε*(*T*) relationship can be described by the Curie–Weiss law (Figure 5). This fact and the characteristic single sharp maximum in the *ε*(*T*) diagrams, as well as the fact that Curie–Weiss temperature is located below the Curie temperature, prove that the obtained ceramic material has a classical phase transformation.

The adjustment of the Curie–Weiss law to the experimental data obtained allowed to determine the Curie temperature (*T_C_*), the Curie–Weiss temperature (*T*_0_) and the Curie constant (*C*) of the BLTF ceramics, which are summarized in Table 2, together with the basic dielectric parameters of the BLTF ceramic.

The increasing concentration of Fe^3+^ ions in the base material results in a significant reduction of the Curie constant and a slight shift of the Curie temperature point towards lower temperatures (from 125 °C for BLTF1 to 120 °C for BLTF4). One of the reasons for the lowering of the phase change temperature may be the influence of the doped ion sizes, and more precisely, their ionic radii. As it is known, in ABO_3_ perovskite ceramic materials the phase transition temperature is proportional to the square of the B ion shift in the oxygen blocker, which can be written as *T_C_* ~ (*δ*_ZB_)^2^. The smaller the B ion in the oxygen octahedron causes lowering of the *δ*_ZB_. Another reason for the phase change shift may be the change in the bandwidth of the *E_g_* band at the Curie temperature and the concentration of electric charge carriers [23].

**Figure 6:**
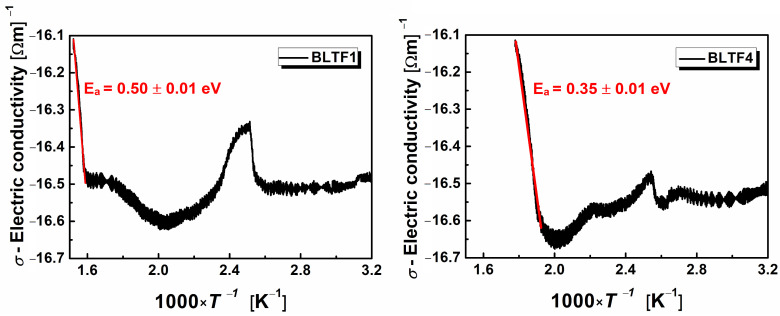
Temperature dependence of conductivity characteristic for BLTF ceramics.

One of the most important electrical properties characterizing engineering materials is electrical conductivity. The total current flowing through the ferroelectric sample in the time of switching consist of the capacitor charging current, the current associated with the occurrence of spontaneous polarization, and the current associated with the electrical volume conductivity. The latter component was measured as a function of temperature for all discussed samples. Examples of ln *σ* (1/*T*) characteristics are shown in Figure 6.

**Figure 7:**
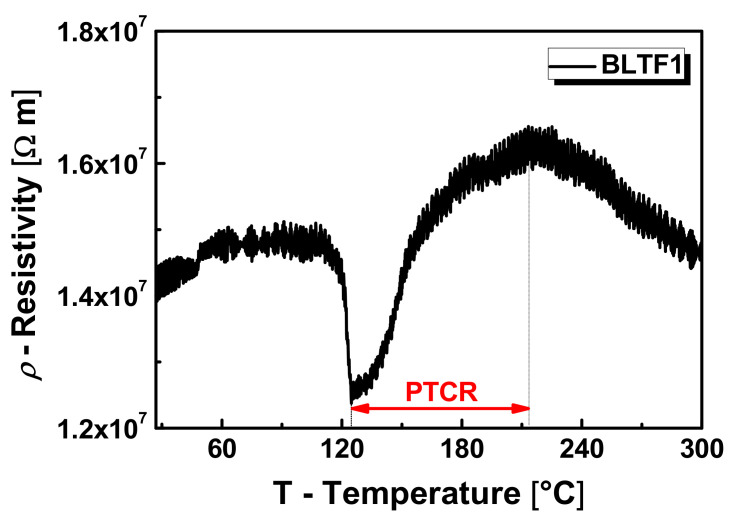
Temperature dependence of conductivity characteristic for BLTF ceramics.

The shapes of the graphs indicate the complexity of the mechanism of electric charge transport in the ceramic materials under study. In the phase transition area, the conductivity reaches its maximum value, then decreases above the *T*_C_ temperature, revealing slight posistor properties—the PTCR effect. Local minima appearing on the dependence of ln *σ* (1/*T*) are related to the polaronic mechanism of transporting electric charges. The minimum value of electrical conductivity may be a result of changing the mechanism of this process from the short-range tunnel to the long-range one the so-called hopping mechanism [24]. The posistor effect is most pronounced in the case of ceramics doped with 0.1 mol.% iron (BLTF1)—Figure 7.

In this case, the temperature coefficient of resistance is *α_T_* = 3.31%/K, and the resistivity Δ*ρ_PTCR_* = 0.42 × 10^7^ Ωm. The temperature range of the PTCR effect for a BLTF1 sample is Δ*T_PTCR_ =* 88 °C. The increase of iron content in the examined ceramic material caused: reduction of the Δ*ρ_PTCR_* to the level of 0.33 × 10^7^ Ωm for BLTF4, reduction of the *α_T_* coefficient to 2.09%/K for BLTF4, and increase of the temperature range of the PTCR effect to 100 °C for BLTF4 (Table 3).

In the range of high temperatures on the dependences of ln*σ* (1/*T*) obtained for tested BLT4 material doped with iron, linear segments are visible, which indicate the activation nature of the conductivity process. The activation energy of this process was determined on the basis of the Arrhenius formula. Its values (*E*_a_) and the temperature range (Δ*T*) were collected in Table 4.

**Figure 8:**
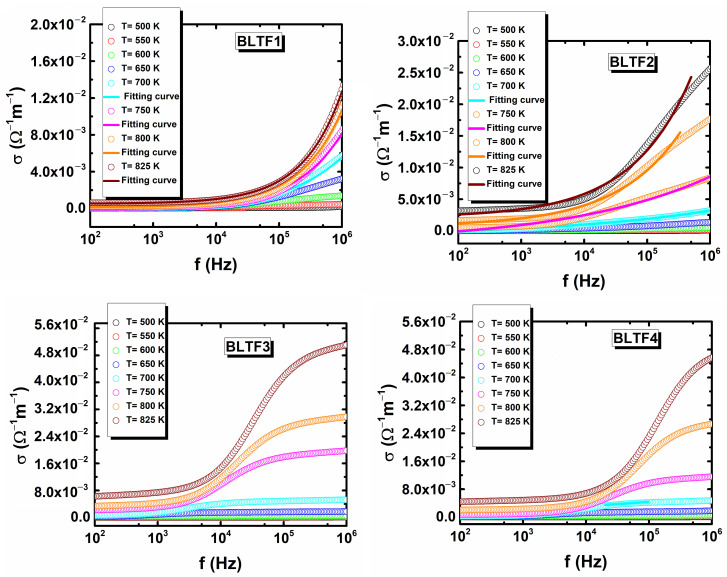
Frequency dependence of AC conductivity of BLTF ceramic at different temperatures.

The minimum temperature of the ln*σ* (1/*T*) function can be equated with the transition temperature between the two aforementioned types of conductivity [24]. Based on information described by other authors [25], the obtained activation energy values indicate that at high temperatures, the electrical conductivity is probably dominated by the ionic conductivity associated with oxygen vacancies, while the hopping polaronic conductivity is activated at low temperatures. These assumptions should be verified by plotting the frequency dependence of AC conduction at different temperatures within the range of 500–850 K. Mentioned characteristics are presented in Figure 8. 

The observed increasing of conductivity with temperature is the normal behavior of the semiconductor materials. The frequency dependence of AC conductivity for two lower concentration of iron ions is comparable with the ones that concern the BaTiO_3_-based materials presented by other authors [26,27,28]. Namely, *σ*_ac_ decreases with decreasing frequency and becomes independent of frequency after a certain value. Extrapolation of this part towards lower frequency will obtain direct current conductivity (*σ*_dc_). The conductivity pattern obeys Jonscher power law:

(1) $$\sigma_{\mathrm{ac}}\left(\omega \right)=\;\sigma_{\mathrm{dc}}+A\omega^{S},$$

where *σ*_dc_ is the frequency independent conductivity which may be obtained by extrapolating the low frequency plateau to zero frequency, A is a constant depending on temperature and determines the strength of polarization, and s is an exponent frequency that lies between 0 and 1. According to the law when a mobile charge carrier jumps to a new position from the old site, it continues in a state of oscillation between two potential energy minima [29,30]. The *σ*_ac_(*f*) dependencies obtained at high temperature for BLTF1 very well fulfill the law. The value of s factor increases from 0.51 to 0.69 when temperature rises from 700 K up to 850 K. The frequency characteristic of *σ*_ac_ obtained for BLTF2 fulfill discussed law only for low frequency. The character of temperature changes of s parameter is similar to the one for BLTF1. The presented above facts could indicate the coexistence of ionic and hopping conductivity in the studied temperature range. The nature of the frequency changes of ac conductivity differ from the next two discussed ceramic materials. Namely, at the range of high frequencies, the value of conductivity is going towards constant values. Such shape of *σ*_ac_(*f*) dependencies does not allow for the adaptation of Joncher’s law, thus it can be assumed that in these materials in the discussed temperature range, the thermal activated ionic conductivity prevails. 

## 4. Conclusions

All discussed samples at room temperature were characterized by single-phased, tetragonal type of structure and the space group *P4mm*. With the increase of Fe^3+^ concentration, reduction of the lattice parameters was stated in result of partial substitution of Ti^4+^ atoms with Fe^3+^ atoms (with a smaller ion radius). These changes affected the physical and functional properties of the material. 

Additionally, the maximum value of dielectric constant in low frequencies was equal to 9814 and 8248 for BLFT1 and BLFT2 ceramics, respectively, and at room temperature was equal to 2339 and 2023, respectively. Those values are significantly lower than the ones for undoped Ba_0.996_La_0.004_Ti_0.999_O_3_. The obtained materials show a classical phase transformation; together with an increase of Fe^3+^ ion concentration, decrease in the value of electric permittivity, as well as the Curie constant and a slight shift of the Curie temperature towards lower values were proved. 

The measurements of temperature changes in DC conductivity revealed that in the phase transition area, the conductivity reaches the maximum value, then decreases above *T_C_* temperature, revealing a slight PTCR effect, which is the strongest for BLTF1 ceramics. An additional advantage of the synthesized materials is the economical and environmentally friendly receiving technology.

## Figures and Tables

**Table 1:** Lattice parameters and cell volume of elementary BLTF ceramics.

Sample	*a*_0_, *b*_0_ [nm]	*c*_0_ [nm]	*V*∙10^−30^ [m^3^]
BLTF1	0.3993(5)	0.4032(2)	64.3
BLTF2	0.3992(7)	0.4031(6)	64.2
BLTF3	0.3993(3)	04031(9)	64.2
BLTF4	0.3993(3)	0.4030(2)	64.2

**Table 2:** Influence of the amount of iron admixture on the BLTF ceramics dielectric parameters: *T_c_*—Curie temperature, *T*_0_—Curie-Weiss temperature, *C*—Curie constant, *ε*—dielectric permittivity at room temperature (Tr), ε_m_—maximum value of dielectric permittivity at *T_c_*, tg*δ*—dielectric loss at *T_r_* and tg*δ* at *T_c_.*

Ceramics	*T_C_* [°C]	*T*_0_ [°C]	*C* [°C]	*ε* at *T_r_* *ν* = 1 kHz	*ε_m_* *ν* = 1 kHz	tg*δ* at *T_r_* *ν* = 1 kHz	tg*δ* at *T_C_* *ν* = 1 kHz
BLTF1	125	113.4	1.366 × 10^5^	2339	9814	0.03	0.01
BLTF2	124	112.3	1.200 × 10^5^	2023	8248	0.02	0.01
BLTF3	124	114.3	0.816 × 10^5^	1005	5766	0.02	0.01
BLTF4	120	106.3	0.647 × 10^5^	1059	3769	0.15	0.01

**Table 3:** Values of posistor parameters of BLTF ceramics.

Research Material	*T_C_* [°C]	*T_PTCRmin_* [°C]	*T_PTCRmax_* [°C]	Δ*T_PTCR_* [°C]	*ρ_min_* [Ωm]	*ρ_max_* [Ωm]	*α_T_* [%/K]
BLTF1	125	125	213	88	1.24 × 10^7^	1.66 × 10^7^	3.31
BLTF2	124	126	218	92	1.33 × 10^7^	1.75 × 10^7^	2.98
BLTF3	124	126	219	93	1.37 × 10^7^	1.74 × 10^7^	2.42
BLTF4	120	120	220	100	1.42 × 10^7^	1.75 × 10^7^	2.09

**Table 4:** The values of the energy activation of the conductivity process and the temperature range of the performed fit for the BLTF ceramics, determined on the basis of studies of temperature changes in the conductivity.

**Ceramics**	**BLTF1**	**BLTF2**	**BLTF3**	**BLTF4**
Δ*T* [K] temperature range	595−567	592−561	578−554	561−526
*E_a_* [eV] activation energy	0.50 ± 0.01	0.40 ± 0.03	0.37 ± 0.02	0.35 ±0.01
